# Bone marrow-derived mesenchymal stem/stromal cells in patients with acute myeloid leukemia reveal transcriptome alterations and deficiency in cellular vitality

**DOI:** 10.1186/s13287-021-02444-0

**Published:** 2021-06-26

**Authors:** Leisheng Zhang, Ying Chi, Yimeng Wei, Wenxia Zhang, Fuxu Wang, Lei Zhang, Linglin Zou, Baoquan Song, Xing Zhao, Zhongchao Han

**Affiliations:** 1 State Key Laboratory of Experimental Hematology & National Clinical Research Center for Blood Disease, Institute of Hematology & Blood Diseases Hospital, Chinese Academy of Medical Sciences & Peking Union Medical College, 288 Nanjing Road, Tianjin, 300020 China; 2 National Joint Local Engineering Laboratory for Cell Engineering and Biomedicine Technique, Guizhou Province Key Laboratory of Regenerative Medicine, Key Laboratory of Adult Stem Cell Translational Research (Chinese Academy of Medical Sciences), Guizhou Medical University, Guiyang, 550004 China; 3 Precision Medicine Division, Health-Biotech (Tianjin) Stem Cell Research Institute Co., Ltd., Tianjin, 301700 China; 4 Department of Neurosurgery, The First Affiliated Hospital & Qianfoshan Hospital of Shandong First Medical University, Ji-nan, 250014 China; 5 Department of Hematology, The Second Hospital of Hebei Medical University, Shijiazhuang, 050000 China; 6 Department of oncology, Affiliated Hospital of Southwest Medical University, Luzhou, 646000 China; 7 National Clinical Research Center for Hematologic Diseases, Jiangsu Institute of Hematology, The First Affiliated Hospital of Soochow University, Suzhou, 215006 China

**Keywords:** Acute myeloid leukemia (AML), BM-MSCs, Cellular vitality, Genomic variation, JAK-STAT signaling

## Abstract

**Background:**

State-of-the-art advances have indicated the pivotal characteristics of bone marrow-derived mesenchymal stem/stromal cells (BM-MSCs) in hematopoietic microenvironment as well as coordinate contribution to hematological malignancies. However, the panoramic view and detailed dissection of BM-MSCs in patients with acute myeloid leukemia (AML-MSCs) remain obscure.

**Methods:**

For the purpose, we isolated and identified AML-MSCs together with healthy donor-derived HD-MSCs from the bone marrow mononuclear cells (BM-MNCs) by using the standard density gradient centrifugation based on clinical diagnosis and cellular phenotypic analysis. Subsequently, we systematically compared the potential similarities and discrepancy both at the cellular and molecular levels via flow cytometry, multilineage differentiation, chromosome karyotyping, cytokine quantification, and transcriptome sequencing and bioinformatic analysis including single-nucleotide polymorphism (SNP), gene ontology (GO), HeatMap, principal component analysis (PCA), Kyoto Encyclopedia of Genes and Genomes (KEGG), and gene set enrichment analysis (GSEA).

**Results:**

On the one hand, AML-MSCs exhibited undistinguishable signatures in cytomorphology, surface biomarker expression pattern, stemness, chromosome karyotype, and chondrogenesis as HD-MSCs, whereas with impaired adipogenesis, enhanced osteogenesis, and variations in cytokine expression pattern. On the other hand, with the aid of genomic and bioinformatic analyses, we verified that AML-MSCs displayed multidimensional discrepancy with HD-MSCs both in genome-wide gene expression profiling and genetic variation spectrum. Simultaneously, the deficiency of cellular vitality including proliferation and apoptosis in AML-MSCs was largely rescued by JAK-STAT signaling inhibition.

**Conclusions:**

Overall, our findings elucidated that AML-MSCs manifested multifaceted alterations in biological signatures and molecular genetics, and in particular, the deficiency of cellular vitality ascribed to over-activation of JAK-STAT signal, which collectively provided systematic and overwhelming new evidence for decoding the pathogenesis of AML and exploring therapeutic strategies in future.

**Supplementary Information:**

The online version contains supplementary material available at 10.1186/s13287-021-02444-0.

## Background

Acute myeloid leukemia (AML), a paradigm of myeloid disorder with multiple life-threatening complications, is characterized by a reduction of physiological differentiation of hematopoietic stem cells (HSCs) towards lymphoid and myeloid lineages in parallel with abnormal activation of pathological hematopoiesis dominated by the accumulation of dysfunctional leukemic blast populations [1–3]. Patients with AML usually accompanied with diverse clonal transformations and pancytopenia in both the bone marrow and blood circulation attribute to the abnormally increased noneffective myeloid progenitor cells and impaired microenvironmental components [4]. Simultaneously, despite the dramatic progress has been made both in exploring the pathogenesis and developing advanced targeted therapies, the patients are still enduring immunedysregulation and the outcomes are far from satisfaction largely due to the deficiency of systematic and intensive investigation of the phenotypic and genetic alterations in AML patients [2, 5].

Since the first isolation and identification in the 1960s, mesenchymal stem/stromal cells (MSCs) have been recognized as the predominant component in the microenvironment and play a pivotal role in physiological hematopoiesis and hematologic malignancies [6–8]. The heterogenous cell population possesses unique characteristics such as hematopoietic-supporting and immunoregulation as well as multilineage differentiation potential towards adipocytes, osteoblasts, and chondrocytes [9, 10]. For decades, we and other investigators have reported the therapeutic effects in recurrent and refractory disorders such as aplastic anemia, acute-on-chronic liver failure (ACLF), critical limb ischemia (CLI), type 2 diabetes, acquired aplastic anemia, and corona virus disease 2019 (COVID-19)-associated acute respiratory distress syndrome (ARDS) [11–17]. Of the abovementioned disorders, blood malignancy and hematological abnormality have obtained broader concerns for MSC-based cytotherapy [6, 7, 12]. However, as to hematologic malignancy like AML, the systematic and detailed characteristics as well as their effects in cancer therapy largely remain obscure over a long period of time, and in particular, the exact cognition of investigations upon tumor-promoting or tumor-suppressing effects of MSCs is inconclusive and even controversial in the context of inpatients with pre-existing hematologic malignancies [18]. Recently, we and Gholizadeh-Ghaleh Aziz et al. reported the MSC-based cancer cell therapy via the engineered (e.g., CD3/CD19 for B cell lymphoma) and nonengineered (e.g., amniotic fluid-derived-MSCs) approaches, respectively [6, 7].

In recent years, state-of-the-art updates are concerned about the underlying rationale of MSC-based therapeutics and pathogenesis-associated multifaceted variations both at the cellular and molecular levels [16, 17, 19]. For instance, Geyh et al. reported the deficiency of AML-MSCs including growth and osteogenic differentiation as well as the insufficient hematopoietic-supporting property, which was associated with a specific methylation signature of Jagged 1 signaling [1]. Therewith, von der Heide et al. conducted the whole exome sequencing (WES) of BM-MSCs derived from AML patients and revealed the global changes in the pattern of molecular alterations [4]. However, to our knowledge, the spectrum of biofunction and molecular complexity between AML-MSCs and HD-MSCs is still obscure, and there is an urgency of dissecting the potential variations together with the underlying pathogenesis in AML-MSCs.

In this study, we conducted multifaceted comparisons upon the biological variations and genetic alterations between AML-MSCs and HD-MSCs both at the cellular and molecular levels. Generally, compared with HD-MSCs, AML-MSCs showed similarities in immunophenotype and G-banded karyotype, but with distinguishable signatures in multilineage differentiation, declined cellular vitality, abnormally activated JAK-STAT signal, and multidimensional discrepancy in gene expression profiling. Furthermore, the dominating deficiency in cellular vitality of AML-MSCs could be largely rescued by JAK-STAT signal inhibition.

## Methods

### Flow cytometry (FCM) assay

The procedure of FCM was conducted as we recently described [9, 12, 20]. In brief, the cultured AML-MSCs and HD-MSCs at passages 3–8 were detached by 0.25% Trypsin/EDTA (Gibco) after reaching 80–90% confluence. After harvesting and washing with 1×PBS (Solarbio) for twice, the indicated MSCs were labelled in dark with fluorescence conjugated monoclonal antibodies, including CD44, CD73, CD90, CD105, CD31, CD34, CD45, and HLA-DR for 30 min. The detailed information of antibodies was listed in Additional file 1: Additional Information: Additional Table S1.

### Quantitative real-time polymerase chain reaction (qRT-PCR) assay

The relative expression levels of indicated mRNAs were verified by qRT-PCR assay as we reported before [6, 21, 22]. Briefly, AML-MSCs and HD-MSCs at indicated timepoints were washed with 1×PBS for twice and lysed with TRIzol reagent (Invitrogen) according to the manufacturer’s constructions [19]. The mRNAs were quantified and synthesized into cDNA with the TransScript Fly First-Strand cDNA Synthesis SuperMix kit (TransGen Biotech). Then, qRT-PCR was conducted by utilizing the ABI PRISM 7900 (Applied Biosystems) and SYBR Green PCR Master Mix kit (Qiagen) as we reported recently [9]. The primer sequences were listed in Additional file 1: Additional Information: Additional Table S2.

### Patients

Blood samples were collected from 37 AML patients (male: 20; female: 17; age: 15–74 years) and 20 HDs (male: 13; female: 7; age: 19–67 years). BM samples were extracted from 3 patients with moderate or high-risk type of AML (male: 2; female: 1; age: 28–35 years) and 3 HDs (male: 2; female: 1; age: 27–31 years). All patients or their patient (legal guardian for patients under 16) and HDs signed informed consents according to the guideline of the Declaration of Helsinki (ethics number: KT2019048-EC-1, 2020-R205). All patients or their patient (legal guardian for patients under 16) were confirmed according to the Guidelines for Clinical Diagnosis of AML. The detailed information of all AML patients or their patient (legal guardian for patients under 16) and HDs was listed in Additional file 2: Additional Table S3, and Additional file 3: Additional Table S4.

### Cell culture

The bone marrow samples from both AML patients (AML-MSCs) and healthy donors (HD-MSCs) for bone marrow-derived MSC (BM-MSC) isolation and identification were provided by the National Clinical Research Center for Blood Disease, Institute of Hematology & Blood Diseases Hospital, Chinese Academy of Medical Sciences & Peking Union Medical College and Department of Hematology in The Second Hospital of Hebei Medical University. AML-MSCs and HD-MSCs were isolated from bone marrow mononuclear cells (BM-MNCs) by utilizing the standard Ficoll (Type 400, Sigma-Aldrich)-based density gradient centrifugation and preserved by our laboratory. The indicated AML-MSCs and HD-MSCs were maintained in DMEM-F12 basal medium (Hyclone) supplemented with 10% FBS (Gibco), 1% GlutaMAX (Gibco), 1% l-glutamine (Sigma), 100 U/ml penicillin/streptomycin (ThermoFisher), 2 ng/ml bFGF (Peprotech), and 10 ng/ml EGF (Peprotech). The culture medium was changed every 3 days. The indicated MSCs were maintained in 37°C, and 5% CO_2_ as we previously reported [10, 12, 14].

### Karyotypic analysis

The genomic stability of AML-MSCs and HD-MSCs was monitored by the G-banded chromosome karyotype analysis. In brief, the aforementioned MSCs in metaphase were treated with colchicine (Tocris) and photographed with an Olympus DA71 microscope (Tokyo) as we recently reported [9, 12, 23].

### Multilineage differentiation of MSCs

Multilineage differentiation potential of AML-MSCs and HD-MSCs was identified as we reported [12, 14, 17]. In brief, 5×10^4^ cells were seeded into 12-well plate wells in MSC culture medium for 2–3 days. After reaching 60–80% confluence, the indicated AML-MSCs and HD-MSCs were cultured in adipogenic-, osteogenic-, and chondrogenic-differentiation medium (Stem Cell Technologies) according to the manufacturer’s instructions, respectively. After 3 weeks’ induction, the MSC-derived adipocytes, osteoblasts, and chondrocytes were identified by Oil Red O staining, Alizarin Red S staining, and Alcian Blue staining, respectively.

### Cell proliferation-associated CCK-8 analysis

The proliferative potential of AML-MSCs and HD-MSCs was analyzed with Cell Counting Kit 8 (Dojindo) as we previously described [9]. Briefly, the indicated MSCs were seeded in MSC culture medium and detected at indicated timepoints according to the manufacturer’s instructions under absorbance at 450 nm (A450). Finally, the curve of population doubling (Pd) was built based on the A450 value.

### Apoptosis assay

The percentage of apoptotic cell population in AML-MSCs and HD-MSCs was determined as we previously reported with several modification [12, 16]. Briefly, the harvested MSCs were pretreated with precooled 1×PBS and 200 μl binding buffer. Then, the MSCs were incubated with the Annexin V Apoptosis Detection Kit (BD) according to the manufacturer’s instructions and the proportion of apoptotic cells were detected by flow cytometry (BD).

### RNA-SEQ and bioinformatic analysis

RNA-SEQ samples were prepared as we previously described with several modification [9, 12, 16]. In details, total mRNAs were extracted from AML-MSCs (passage 3, *n* = 3) (Additional file 2: Additional Table S3) and HD-MSCs (passage 3, *n* = 3) (Additional file 3: Additional Table S4) by utilizing TRIzol reagent (Invitrogen) according to the manufacturer’s instructions and qualified by NanoDrop (Thermo). Then, the RNAs were sequenced by the Novogene (Tianjin, China). The bioinformatic analyses were performed as we recently reported according to the instructions of the databases and online platforms [9, 10, 12]. The differentially expressed genes were listed in Additional file 4: Additional Table S5, and Additional file 5: Additional Table S6.

### Statistical analysis

All statistical analyses were performed with Prism 6.0 (GraphPad Software) as we reported before [10, 17, 24, 25]. In brief, the comparison of two different unpaired groups was analyzed with unpaired *t* test, while analysis among multiple unpaired groups was conducted with one-way ANOVA test. The statistically significant difference was considered only when *P* value was less than 0.05. All data were shown as mean ± SEM (*n* = 3 independent experiments). *P < 0.05; **P < 0.01; ***P < 0.001; NS, not significant.

## Results

### AML-MSCs showed similarities in immunophenotype and chromosome karyotype with HD-MSCs

**Fig. 1:**
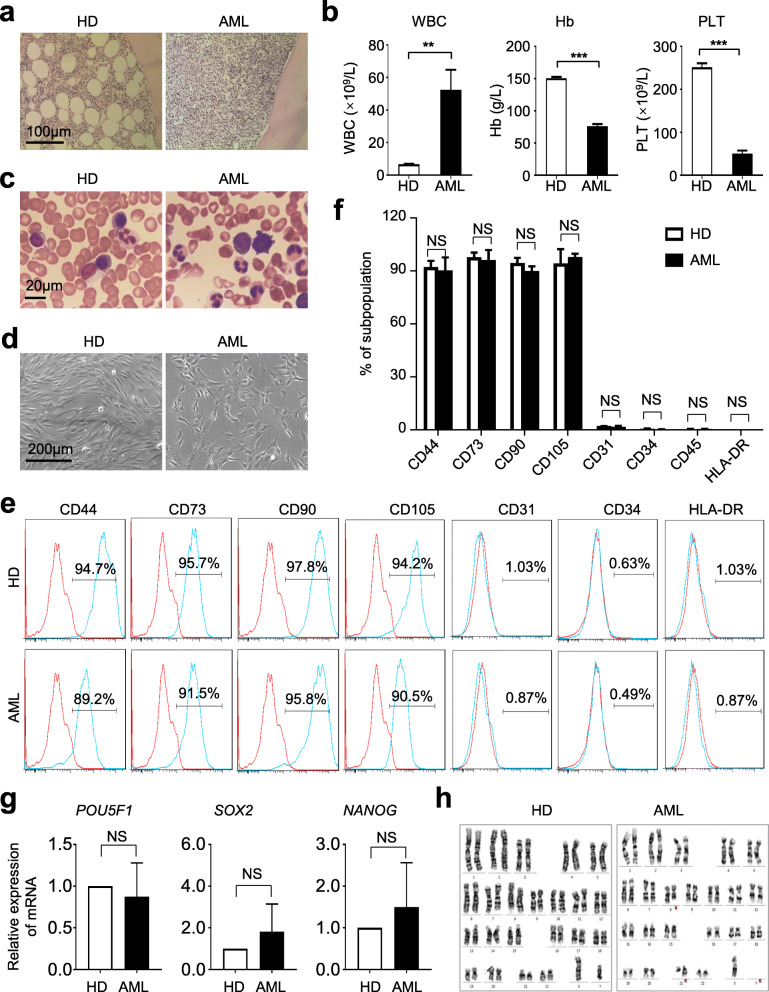
AML-MSCs and HD-MSCs showed similarities in immunophenotyping and stemness. **a** Representative clinicopathologic sections of bone morrow from healthy donors (HD) and AML patients (AML), respectively. Scale bar=100 μm. **b** Bone marrow biopsy of hemocytes in HD and AML patients, respectively. Scale bar=20 μm. **c** Blood routine examination of peripheral blood (WBC, Hb, PLT) in HD and AML. **d** Representative morphogen of cultured bone marrow-derived MSCs (HD-MSCs, AML-MSCs). **e**, **f** Representative histogram (**e**) and statistical analysis (**f**) of biomarker expression in HD-MSCs and AML-MSCs by flow cytometry (FCM). Data were shown as mean ± SEM (*n* = 3). NS, not significant. **g** qRT-PCR analysis of stemness-associated (*POU5F1*, *SOX2*, *NANOG*) biomarkers in HD-MSCs and AML-MSCs (mean ± SEM, *n* = 4). NS, not significant. **h** Karyotypic analysis of AML-MSCs and HD-MSCs

Over the years, the latest progress of mesenchymal stem/stromal cells (MSCs) in the bone marrow for physiological and pathological hematopoiesis and immunoregulation has come into the spotlight [12, 23]. However, multifaceted characterization of the cellular and genetic signatures of MSCs in acute myeloid leukemia (AML) is far from satisfaction [1, 4, 26]. For the purpose, we primitively analyzed a cohort of 37 AML patients and 20 healthy donors (HD) by conducting clinicopathological examination of bone marrow biopsy as well as analyzing the components of blood samples (Fig. 1a–c). Of them, 3 representative AML patients and coordinate HDs were enrolled for further analyses. In consistence with previous reports, both HD-MSCs and AML-MSCs isolated from the bone marrow showed typical spindle-like morphogen whereas AML-MSCs appeared a little more swollen and spreading (Fig. 1d). Furthermore, immunophenotypic analysis indicated that the aforementioned MSCs with indistinguishable surface biomarker expression pattern (Fig. 1e–f). Interestingly, compared with those in the HD-MSCs, we found AML-MSCs revealed minimal differences in pluripotency-associated gene expression and G-banded chromosome karyotype, which collectively suggested that AML-MSCs had little correlation with stemness impairment and chromosome abnormalities at the genomic level (Fig. 1g–h).

### AML-MSCs exhibited diverse alterations in multilineage differentiation and cytokine expression

**Fig. 2:**
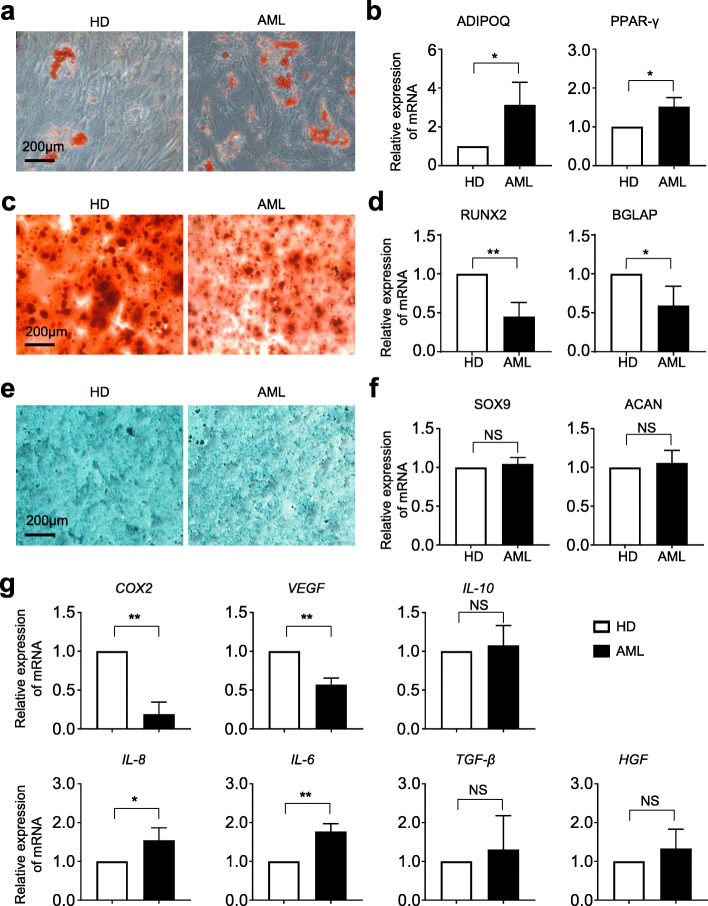
Comparation of multilineage differentiation potential and cytokine expression in HD-MSCs and AML-MSCs. **a**, **b** Adipogenic differentiation potential of HD-MSCs and AML-MSCs identified by Oil Red O staining (**a**) and qRT-PCR analysis of adipogenic differentiation-associated genes (*ADIPOQ*, *PPAR-γ*) (**b**). **c**, **d** Osteogenic differentiation potential of HD-MSCs and AML-MSCs identified by Alizarin Red S staining (**c**) and qRT-PCR analysis of osteogenic differentiation-associated genes (*RUNX2, BGLAP*) (**d**). **e**, **f** Chondrogenic differentiation potential of HD-MSCs and AML-MSCs identified by Alician Blue staining (**e**) and qRT-PCR analysis of chondrogenic differentiation-associated genes (*SOX9*, *ACAN*) (**f**). **g** Quantitative analysis of representative cytokines and inflammatory factors (*COX2*, *VEGF*, *IL-10*, *IL-8*, *IL-6*, *TGF-β*, *HGF*) in HD-MSCs and AML-MSCs. Scale bar=200 μm. All data were shown as mean ± SEM (*n* = 4). *P < 0.05; **P < 0.01; NS, not significant

To further verify the potential variations in biological phenotypes, we conducted multilineage differentiation analysis upon the abovementioned AML-MSCs and HD-MSCs. As shown by Oil Red O staining, more adipocytes were generated in the AML-MSC group, which was confirmed by qRT-PCR analysis of adipogenic differentiation-associated genes (Fig. 2a, b). Diametrically, compared with the HD-MSC group, decreased areas with Alizarin Red S staining and declined expression of osteoblast-associated biomarkers were observed in AML-MSCs after a 3-week osteogenic differentiation (Fig. 2c, d). However, as to chondrogenic differentiation, there were no statistically significant differences between HD-MSCs and AML-MSCs in Alician Blue staining and chondrogenic differentiation-associated gene expression, respectively (Fig. 2e, f). Thereafter, considering that secreted factors might involve in mediating AML-MSC dysfunction, we turned to quantitative analysis and found that a spectrum of intrinsic proinflammatory- and inflammatory-associated cytokines exhibited diverse alterations in expression as well (Fig. 2g).

### AML-MSCs revealed multifaceted discrepancy in gene expression profiling with HD-MSCs

**Fig. 3:**
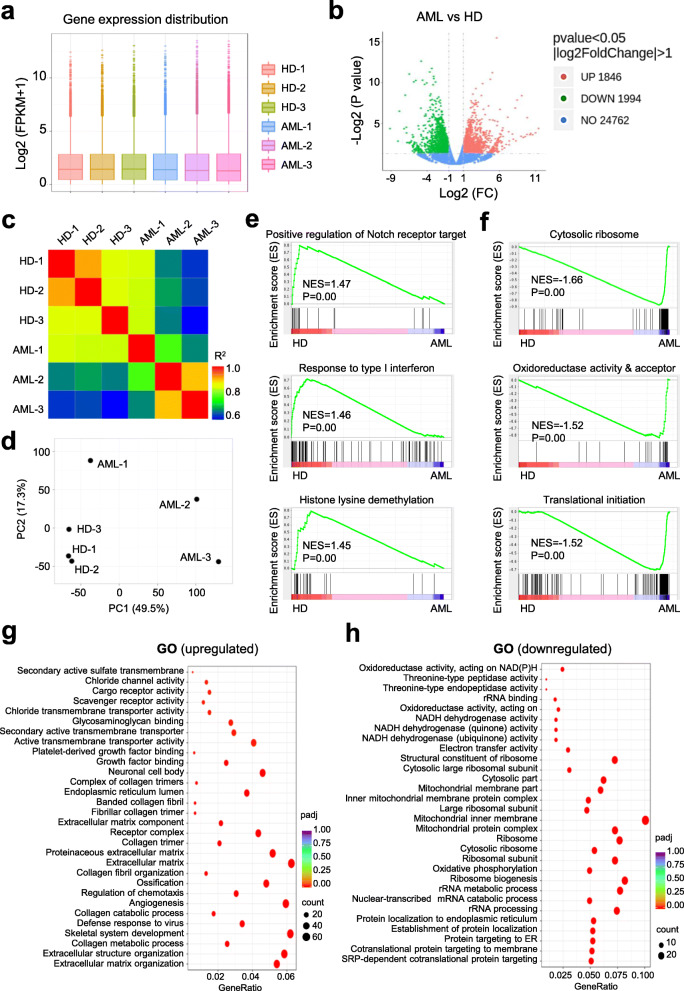
Gene expression profiling of HD-MSCs and AML-MSCs. **a**, **b** The distributions of gene expression in HD-MSCs (HD-1, HD-2, HD-3) and AML-MSCs (AML-1, AML-2, AML-3) based on log_2_ (FPKM+1) (**a**) and -log_2_ (*P* value) (**b**), respectively. **c** Correlation analysis of AML-MSCs and HD-MSCs by HeatMap diagram. **d** Principal component analysis (PCA) of AML-MSCs and HD-MSCs. **e**, **f** Gene set enrichment analysis (GSEA) of differentially functional gene subsets in HD-MSCs (**e**) and AML-MSCs (**f**), respectively. **g**, **h** Gene ontology of upregulated genes (**g**) and downregulated genes (**h**) in AML-MSCs compared with HD-MSCs

Having clarified the cellular properties of AML-MSCs and HD-MSCs, we were curious about the potent similarities and differences at the molecular level. With the aid of high-throughput sequencing, we found that the general distribution of the total fragments per kilobase million (FPKM) values showed similarities and merely differences, whereas the individual FPKM values were different amongst the 3 HD-MSCs and 3 AML-MSCs (Fig. 3a, Additional file 4: Additional Table S5, and Additional file 5: Additional Table S6). Conversely, we found a total number of 1846 and 1994 genes were significantly upregulated and downregulated (*P* < 0.05, |log_2_FC|>1) in AML-MSCs compared with HD-MSCs, respectively (Fig. 3b). As expected, the indicated 3 HD-MSCs or 3 AML-MSCs respectively manifested closer clustering characteristics in gene expression pattern as confirmed by HeatMap diagram and principal component analysis (PCA) (Fig. 3c, d). Simultaneously, by conducting gene set enrichment analysis (GSEA), we figured out that certain datasets, including positive regulation of notch receptor targets, response to type I interferon, and histone lysine demethylation, were collectively enriched in the HD-MSC group, while subsets such as cytosolic ribosome, oxidoreductase activity and acceptor, and translational initiation were representatively enriched in AML-MSCs (Fig. 3e, f). Furthermore, with the aid of gene ontology (GO) analysis of differentially expressed genes, we noticed those upregulated ones in AML-MSCs were involved with extracellular matrix, angiogenesis, and growth factor binding. Instead, the significantly downregulated genes in AML-MSCs were associated with multiple metabolic processes including rRNA metabolic process, ribosomal subunit, mitochondrial membrane, and oxidative phosphorylation (Fig. 3g, h). Taken together, these data suggested the multifaceted alterations in the spectrum of gene expression in AML-MSCs as well as the coordinate dysfunctions.

### AML-MSCs manifested distinguishable signatures of genetic variation spectrum and gene interaction network

**Fig. 4:**
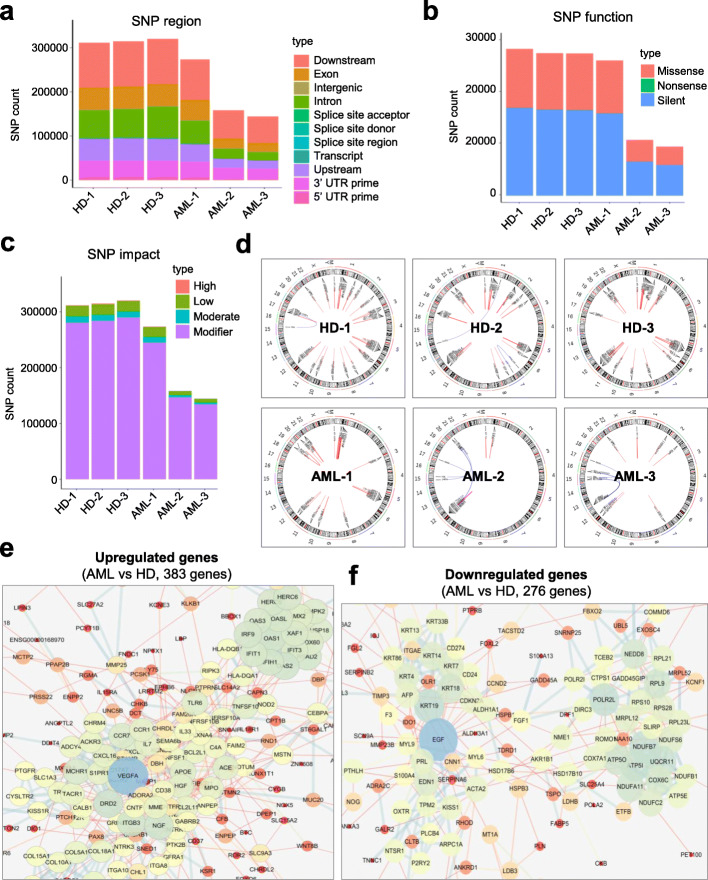
The variation of SNP spectrum and interaction of differentially expressed genes between AML-MSCs and HD-MSCs. **a**, **c** The variations in the distribution (**a**), amount (**b**) and impact (**c**) of single-nucleotide polymorphism (SNP) in AML-MSCs (*n* = 3) and HD-MSCs (*n* = 3). **d** The variations of loci regional distribution of SNP variations and gene fusion events were verified by Circos software. **e**, **f** The interaction of highly expressed genes in AML-MSCs (**e**) and HD-MSCs (**f**), respectively

With the aim of further illuminating the potential abnormalities at molecular level, we meticulously compared the genetic modifications of AML-MSCs with those of HD-MSCs. Initiatively, by dissecting the distribution of single-nucleotide polymorphism (SNP), we found the amount of SNP regions were sharply decreased in AML-MSCs compared with that in HD-MSCs (Fig. 4a). In accordance, the subsets of SNP function (missense, silent), together with the degrees of SNP impact (high, modifier), were notably declined in AML-MSCs as well (Fig. 4b, c). Furthermore, as shown by the Circos analysis, the somatic variations and expressions including SNPs, INDELs, gene fusion events, and FPKM values as well as their loci regional distributions could be intuitively observed in the chromosomes of AML-MSCs and HD-MSCs (Fig. 4d). Finally, to clarify the potential alterations in the protein interaction network between the aforementioned MSCs, we took advantage of DIAMOND and STRING database and found that the 383 upregulated DEGs (e.g., VEGFA, DRD2, ACE, IRF9) and 276 downregulated ones (e.g., EGF, IDO1, POLR2L, NDUFB7) were closely related to disparate protein clusters, respectively (Fig. 4e, f).

### Deficiency of cellular vitality in AML-MSCs was largely rescued by JAK-STAT signaling inhibition

**Fig. 5:**
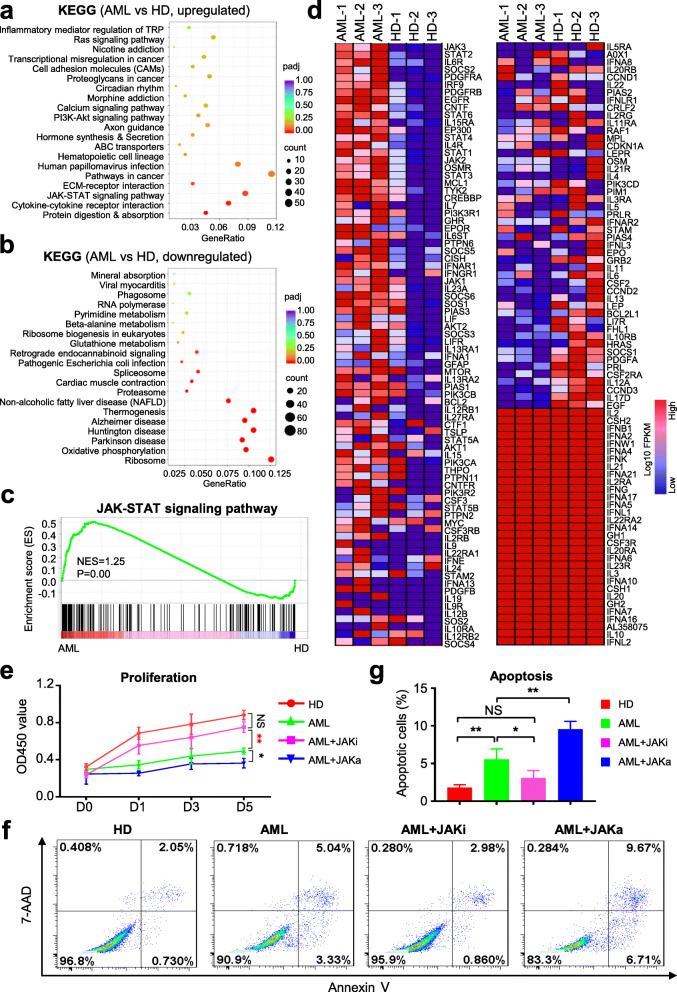
JAK-STAT signaling inhibition was sufficient to counteract defects of cellular vitality of AML-MSCs. **a**, **b** The hyperactivated (**a**) and extensive inhibited (**b**) genes were involved in multiple signaling pathways in AML-MSCs compared with those in HD-MSCs by KEGG analysis. **c** GSEA indicated the differentially expressed genes were involved in JAK-STAT signaling pathway subsets in AML-MSCs. **d** The HeatMap diagram of JAK-STAT signaling pathway-associated genes in AML-MSCs and HD-MSCs. **e**, **g** The proliferation ability (**e**) and apoptotic rate (**f**, **g**) of HD-MSCs and AML-MSCs with/without JAK-STAT signal inhibition (JAKi) or activation (JAKa). All data were shown as mean ± SEM (*n* = 3). *P < 0.05; **P < 0.01; NS, not significant

To evaluate the underlying alterations accompanied with differentially expressed genes (DEGs), we turned to signal analysis by utilizing the Kyoto Encyclopedia of Genes and Genomes (KEGG) pathway database. As shown by the diagram, the PI3K-Akt signaling pathway was abnormally activated in AML-MSCs, which was consist with current reports in leukemogenesis [27]. Simultaneously, we noticed that JAK-STAT signaling pathway, together with inflammation, transcriptional misregulation, pathways in cancer, and cytokine-cytokine receptor interaction associated signals, was collectively enriched in AML-MSCs (Fig. 5a). Conversely, compared with the HD-MSC group, those downregulated signaling pathways in AML-MSCs were mainly involved in multiple metabolisms, thermogenesis, ribosome, and diseases (Fig. 5b). Therewith, with the aid of GSEA, we intuitively observed the distribution and enrichment score of JAK-STAT signaling pathway associated genes between AML-MSCs and HD-MSCs (Fig. 5c). Finally, from the overview of the HeatMap diagram, we could intuitively observe the detailed variations of JAK-STAT associated genes, which further confirmed the presumptive hyperactivation of the indicated signaling in AML-MSCs (Fig. 5d).

Simultaneously, we noticed the decreased proliferation of AML-MSCs during in vitro cultivation compared with HD-MSCs, which was in line with Geyh et al. reported [1]. Therefore, we were inquisitive about the potential dysregulation of cellular vitality in AML-MSCs attributed to abnormal activation of JAK-STAT signaling. For the purpose, we originally conducted CCK-8 analysis and found that AML-MSCs were declined in cell proliferation compared to HD-MSCs, which could be almost rescued by adding inhibitors of JAK-STAT signaling (short for JAKi) whereas even worsen by adding activators instead (short for JAKa) (Fig. 5e). Similarly, distinguish from those caused by JAKa, JAKi administration was sufficient to ameliorate the high percentage of abnormally apoptotic cell population in AML-MSCs (5f, g). Collectively, we concluded that appropriate inhibition of JAK-STAT signaling in AML-MSCs could significantly relieve the deficiency of cellular vitality including cell proliferation and apoptosis.

## Discussion

Acute myeloid leukemia (AML) as well as myelodysplastic syndromes (MDS) are representative myeloid disorders with hallmarks of hematopoietic insufficiency and the resultant cytopenia-associated complication [28, 29]. Even though remarkable improvements have been achieved for diagnosis and treatment of AML, yet a certain number of patients are still enduring pancytopenia, bleeding and infections or even HSC transplant failure and rejection, which are also the major causes for mortality and morbidity [1, 28]. State-of-the-art updates upon bone marrow microenvironment have suggested the pivotal role of MSCs in physiological hematopoiesis and coordinate contribution to hematological malignancies [1, 4, 18]. Nevertheless, to our knowledge, systematic and rigorous exploration of the multifaceted variations of MSCs in leukemogenesis as AML-MSCs remains insufficient. For the purpose, in this study, we primitively isolated and identified AML-MSCs from representative AML patients with informed consents. Interestingly, both AML-MSCs and HD-MSCs showed similarities in immunophenotype and chromosome karyotype whereas the former exhibited multidimensional defects in biological functions and genetic variations. Strikingly, the dominating deficiency of cellular vitality in AML-MSCs attributes to hyperactivation of JAK-STAT signaling was largely rescued by clinical inhibitors. In coincidence with the recent studies upon AML-MSCs, our data provided overwhelming evidences for AML-MSCs as environmental pathogenic factors and could help increase knowledge of the pathogenesis and therapeutics of AML.

As the major component in the microenvironment, MSCs have proverbially attracted the attention of the field both in fundamental research and clinical application [1, 12, 16]. On the one hand, an appreciable quantity of investigators concentrates on illuminating the similarities and distinctions of MSCs with different origins such as the bone marrow (BM-MSCs), umbilical cord (UC-MSCs), adipose (AD-MSCs), dental pulpal (DPSC), and even human pluripotent stem cells (hPSC-MSCs), which would be of tremendous assistance for regenerative medicine [10, 20, 30, 31]. On the other hand, the current studies have demonstrated the involvement of MSCs in multiple disease progression including leukemia and coordinated hematological disorders [1, 12]. For instance, we and colleagues recently reported the peripancreatic AD-MSCs from type 2 diabetics with conservative alterations in multidimensional signatures including indistinguishable cellular vitality and stemness, immunophenotype, and chondrogenic differentiation [19]. Conversely, BM-MSCs derived from patients with aplastic anemia (AA-MSCs) exhibited multifaceted defects and variations in biological natures and molecular genetics in the whole genome [12].

As to AML-MSCs, with the aid of scattered functional tests and bioinformatic analysis based on whole-exome sequencing (WES) of MSCs or micro-RNA profiling of MSC-derived exosomes, the pioneers have primitively described the potential distinctions with HD-MSCs both at the cellular and molecular level [4]. In details, Geyh et al. addressed the contribution of AML-MSCs to hematopoietic failure in patients and verified the significant growth deficiency and osteogenic differentiation capacity together with specific methylation signatures affecting skeletal development and stromal support for long-term HSC maintenance, which was subsequently confirmed by TGF-β1-mediated functional inhibition of osteogenic differentiation and hematopoietic support capacity [1, 32]. However, the systematic dissection of the biological phenotypes (e.g., surface biomarkers, stemness, chromosome karyotypes, cytokine expression profile, adipogenic, and chondrogenic differentiation potential) and transcriptomic variations (e.g., gene expression distribution, biological function of differently expressed genes, subtypes and distribution of SNP region, and signaling pathway) in AML-MSCs is largely unknown [4, 28]. Herein, with the aid of functional identification and transcriptomic analyses, we for the first time drew the landscape of AML-MSCs from the view of cellular and molecular signatures, and in particular, the dysfunction of JAK-STAT signal over-activation for the decline in cellular vitality.

Interestingly, with the aim of dissecting genome-wide genetic, transcriptional, and epigenetic alterations associated with the pathogenesis of AML, another report by Baldus et al. revealed a non-specific pattern of genetic alterations in AML-MSCs except the deregulation of proteoglycans and adhesion molecules as well as metabolic pathways and endocytosis [4]. Instead, by conducting transcriptomic analyses, we observed the multifaceted variations in a series of biological processes such as oxidoreductase activity, mitochondrial protein and rRNA processing, regulation of chemotaxis, extracellular matrix organization, histone lysine demethylation, translational initiation, and growth factor binding, which collectively indicated the diversity and complexity of AML-MSCs at molecular level. Furthermore, distinguished from the aforementioned gene expression profiling and epigenetic modification pattern, AML-MSC-derived exosome revealed an abnormal spectrum of miRNA profiling (e.g., upregulated miR-26a-5p and miR-101-3p, downregulated miR-23b-5p, and miR-339-3p) and predicted leukemogenesis-associated gene expression (e.g., upregulated KRBA2 and RRBP1, downregulated EZH2 and GSK3β) [33]. Even though, most of the current studies upon AML-MSCs are relatively piecemeal and the detailed and in-depth cognitive explanation of the pathogenesis for AML is still woefully inadequate. Herein, we systematically clarified the multifaceted variations including similarities in immunophenotype and chromosome karyotype, decreased cellular vitality (declined population doubling and cell cycle, deteriorative apoptosis, and senescence), diverse multilineage differentiation as well as multidimensional alterations in epigenetic modification (e.g., SNPs, INDELs, gene fusion events) and gene expression profiling (DEGs associated with immunedysregulation, synthesis, and metabolism), and interactive network-associated genetic variations (e.g., *VEGFA* and *IFIT3* in AML-MSCs, *EGF*, and *POLR2L* in HD-MSCs). Strikingly, the deficiency of cellular vitality could be vigorously rescued by JAK-STAT inhibitors, which further illuminated the potential pathogenesis and therapeutics in AML. Meanwhile, it is worth noting that the role of telomere and telomerase in various cancers, especially in hematologic malignancies like AML as well as stem cell biology, has been put forward by scholars in the field [34–36]. For instance, Fathi and colleagues recently reported the effect of BM-MSCs upon the reduction of telomerase activity of leukemia cell line, which indicated the therapeutic potential of MSC-derived cytokines (e.g., IL-6, IL-8, TGF-β) for immortality features of hematological tumor cells [37]. Taken together, in conjunction with the aforementioned studies in the field, our data further confirmed the pivotal characteristics and pathogenesis of MSCs in AML and provided overwhelming new references for systematic and rigorous investigation of AML-MSCs as well.

## Conclusions

Overall, in the study, we successfully isolated and identified both AML-MSCs and HD-MSCs, and systematically compared the similarities and differences in biological signatures, cellular vitality, and gene expression pattern. Our studies would supply references for the basic research and clinical applications in future.

## Supplementary Information

**Additional file 1: Additional Information.** The details accompanied with the main manuscript including Additional Table S1-S2 were listed.

**Additional file 2: Additional Table S3.** General information of the AML patients.

**Additional file 3: Additional Table S4.** General information of the HDs.

**Additional file 4: Additional Table S5.** The upregulated genes (HD-MSCs vs AML-MSCs).

**Additional file 5: Additional Table S6.** The downregulated genes (HD-MSCs vs AML-MSCs).

## Data Availability

All data generated or analyzed during this study, together with the additional files, are included in this published article. Meanwhile, the datasets involved in the current study are available from the corresponding author on reasonable request.

## Abbreviations

AML: Acute myeloid leukemia
MSCs: Mesenchymal stem/stromal cells
AD-MSCs: Adipose tissue-derived MSCs
UC-MSCs: Umbilical cord-derived MSCs
BM-MSCs: Bone marrow-derived MSCs
hPSC-MSCs: Human pluripotent stem cell-derived mesenchymal stem/stromal cells
DPSCs: Dental pulpal-derived stem cells
WES: Whole-exome sequencing
AA-MSCs: Aplastic anemia-derived MSCs
HD-MSCs: Healthy donor-derived MSCs
MDS: Myelodysplastic syndromes
SNP: Single-nucleotide polymorphism
KEGG: Kyoto Encyclopedia of Genes and Genomes
GO: Gene ontology
GSEA: Gene set enrichment analysis
PCA: Principal component analysis
FPKM: Fragments per kilobase million
CLI: Critical limb ischemia
COVID-19: Corona virus disease 2019
HSC: Hematopoietic stem cells
