# Supplementary material for: Bone marrow-derived mesenchymal stem/stromal cells in patients with acute myeloid leukemia reveal transcriptome alterations and deficiency in cellular vitality
Source: Stem Cell Res Ther. 2021 Jun 26;12:365. doi: 10.1186/s13287-021-02444-0 (PMC8233618; doi:10.1186/s13287-021-02444-0)
Supplement: Supplementary file 1 — Additional file 1: Additional Information. The details accompanied with the main manuscript including Additional Table S1-S2 were listed. [file 13287_2021_2444_MOESM1_ESM.docx]

**Supplementary Information**

**Bone Marrow-derived Mesenchymal Stem/Stromal Cells in Patients with Acute Myeloid Leukemia Reveal Transcriptome Alterations and Deficiency in Cellular Vitality**

Leisheng Zhang^1-5#*^, Ying Chi^1#^, Yimeng Wei^1^, Wenxia Zhang^5^, Fuxu Wang^5^, Lei Zhang^2^, Linglin Zou^6*^, Baoquan Song^7*^, Xing Zhao^2*^, Zhongchao Han^1,3*^

**Additional Information:**

Additional Tables: Table S1-S2.

**Additional Tables**

**Table S1. Antibodies used in this study.**

Antibodies for flow cytometry assay and immunofluorescent staining.

| Antibody | Cat.NO. | Source |
| --- | --- | --- |
| Anti-CD73-PE | 550257 | BD Pharmingen |
| Anti-CD90-FITC | 561969 | BD Pharmingen |
| Anti-CD105-PE | 560839 | BD Pharmingen |
| Anti-CD31-PE | 560975 | BD Pharmigen |
| Anti-CD34-PE | 560941 | BD Pharmingen |
| Anti-HLA-DR-FITC | 562008 | BD Pharmingen |

**Table S2. Primers used in this study.**

Real-time PCR primer sequences.

| Gene | Forward Primer | Reserve Primer |
| --- | --- | --- |
| *ACTIN* | CTCTTCCAGCCTTCCTTCCT | AGCACTGTGTGTTGGCGTACAG |
| *POU5F1* | CTTGAATCCCGAATGGAAAGGG | GTGTATATCCCAGGGTGATCCTC |
| *SOX2* | GCCGAGTGGAAACTTTTGTCG | GGCAGCGTGTACTTATCCTTCT |
| *NANOG* | TTTGTGGGCCTGAAGAAAACT | AGGGCTGTCCTGAATAAGCAG |
| *ADIPOQ* | TGGTCCTAAGGGAGACATCG | TGGAATTTACCAGTGGAGCC |
| *PPAR-γ* | GCTGGCCTCCTTGATGAATA | TGTCTTCAATGGGCTTCACA |
| *RUNX2* | CTCACTACCACACCTACCTG | TCAATATGGTCGCCAAACAGATTC |
| *BGLAP* | GGCGCTACCTGTATCAATGG | TCAGCCAACTCGTCACAGTC |
| *ACAN* | CCCCTGCTATTTCATCGACCC | GACACACGGCTCCACTTGAT |
| *SOX9* | AATGGAGCAGCGAAATCAAC | CAGAGAGATTTAGCACACTGATC |
| *IL-6* | ACTCACCTCTTCAGAACGAATTG | CCATCTTTGGAAGGTTCAGGTTG |
| *IL-8* | CTGCGCCAACACAGAAATTA | TGAATTCTCAGCCCTCTTCAA |
| *IL-10* | TCACATGCGCCTTGATGTCTG | GATGTCAAACTCACTCATGGCT |
| *TGF-β* | GGCCAGATCCTGTCCAAGC | GTGGGTTTCCACCATTAGCAC |
| *HGF* | GCTATCGGGGTAAAGACCTACA | CGTAGCGTACCTCTGGATTGC |
| *COX-2* | ACTCTGGCTAGACAGCGTAA | ACCGTAGATGCTCAGGGAC |
